# Wheat Water-Soluble Carbohydrate Remobilisation under Water Deficit by *1-FEH w3*

**DOI:** 10.3390/cimb45080419

**Published:** 2023-08-11

**Authors:** Nusrat Khan, Jingjuan Zhang, Shahidul Islam, Rudi Appels, Bernard Dell

**Affiliations:** 1 Centre for Crop and Food Innovation, Food Futures Institute, Murdoch University, 90 South Street, Murdoch, WA 6163, Australia; nusrat.khan@ndsu.edu (N.K.); j.zhang@murdoch.edu.au (J.Z.); shahidul.islam.1@ndsu.edu (S.I.); 2 Department of Plant Sciences, North Dakota State University, Fargo, ND 58102, USA; 3 Faculty of Science, University of Melbourne, Parkville, VIC 3010, Australia; rudiappels5@gmail.com

**Keywords:** *1-FEH* isoforms, gene mutation, water deficit, water-soluble carbohydrate, wheat

## Abstract

Fructan 1-exohydrolase (1-FEH) is one of the major enzymes in water-soluble carbohydrate (WSC) remobilisation for grains in wheat. We investigated the functional role of *1-FEH w1*, *w2*, and *w3* isoforms in WSC remobilisation under post-anthesis water deficit using mutation lines derived from the Australian wheat variety Chara. F1 seeds, developed by backcrossing the *1-FEH w1*, *w2*, and *w3* mutation lines with Chara, were genotyped using the Infinium 90K SNP iSelect platform to characterise the mutated region. Putative deletions were identified in *FEH* mutation lines encompassing the *FEH* genomic regions. Mapping analysis demonstrated that mutations affected significantly longer regions than the target *FEH* gene regions. Functional roles of the non-target genes were carried out utilising bioinformatics and confirmed that the non-target genes were unlikely to confound the effects considered to be due to the influence of *1-FEH* gene functions. Glasshouse experiments revealed that the *1-FEH w3* mutation line had a slower degradation and remobilisation of fructans than the *1-FEH w2* and *w1* mutation lines and Chara, which reduced grain filling and grain yield. Thus, *1-FEH w3* plays a vital role in reducing yield loss under drought. This insight into the distinct role of the *1-FEH* isoforms provides new gene targets for water-deficit-tolerant wheat breeding.

## 1. Introduction

Water deficit during grain filling reduces photosynthesis and increases the dependency of wheat crops on water-soluble carbohydrates (WSC) stored in the stem for grain filling. Wheat fructans, a major component of WSC, must be depolymerised (hydrolysed) before remobilisation to the grain, and this is regulated by a class of enzymes called fructan exohydrolases (FEHs) [1]. So far, two major types of FEHs have been characterised and cloned: 1-FEHs that preferentially degrade inulin-type fructans having *β*-(2,1) linkage [2,3] and 6-FEHs that degrade levan-type fructans with *β*-(2,6) linkage [2,4,5].

1-FEH catalyses fructan breakdown and is considered a *β*-(2,1)-trimmer during active fructan biosynthesis [2]. Elevated 1-FEH activities lead to the breakdown of fructan, thus releasing more sucrose and fructose when the demand for sucrose is high for grain filling [2,6,7,8,9]. Three isoforms of the *1-FEH* gene have been reported, namely *1-FEH w1* (*1-FEH*-6A), *1-FEH w2* (*1-FEH*-6D), and *1-FEH w3* (*1-FEH*-6B). Previous findings from our laboratory suggested that the dominant expression of *1-FEH w3* might facilitate higher fructan remobilisation under terminal water deficit [10]. However, the individual functions of the three isoforms are still unknown due to the complex nature of the plant’s response to water-deficit stress.

Knockout mutation lines of each of the three isoforms (*1-FEH w1*, *w2*, and *w3*) were developed by heavy ion bombardment at CSIRO, Canberra, Australia, in the background of Chara [11], which is a moderately water-deficit-tolerant bread wheat cultivar. Mutating particular gene/s of interest is a common genetic approach to studying biological phenomena and defining gene function [12]. Knocking out genes is a straightforward method of producing gene mutation lines, and this approach has been used in many studies [13]. While mutation lines are essential for forward genetic studies of gene function, recent advancement in sequencing the wheat genome [14] also enables their application in reverse genetics. However, in polyploidy plant species like wheat, which contain multiple copies of the same gene (homologous gene) encoded by each of the ancestral genomes, mutational gene inactivation/deletion is very important to determine whether different copies of the same or related genes are functionally active or redundant [11].

The use of mutational gene inactivation/deletion in polyploid plant species is complicated [15], and for wheat, the challenge is that it possesses three ‘subgenomes’, designated ‘A’, ‘B’, and ‘D’, which are approximately 17 Gb in total size and 40 times the size of the rice genome. In addition to its large size, the wheat genome contains a high percentage of repetitive segments (80–90%) [16,17]. Generally, most wheat genes have three homoeologous copies on the A, B, and D subgenomes, leading to functional redundancy [15,18]. This gene redundancy causes ambiguities in gene knockout strategies, and, as a result, wheat is tolerant of mutations. Although such tolerance to mutation is generally desirable to keep the overall phenotype functional through the generations, genomic redundancy is challenging for producing loss-of-function mutants for a gene of interest [19]. Moreover, heavy ion bombardment causes different mutation types, including base substitutions, deletions, and chromosomal alteration [20,21]. Therefore, it is essential to investigate how gene inactivation works, for example, whether inactivation is by DNA sequence alterations or complete deletion and whether the mutation has caused any polymorphism in the coding sequence or an indirect modification of gene function. Most importantly, any closely linked genes, which might influence WSC remobilisation, need to be identified.

Whole-genome sequencing is a straightforward and powerful tool to directly identify mutagen-induced nucleotide changes linked to the causal mutation and recognise specific genomic locations of the mutated region [22]. High-density SNP arrays have been optimised and successfully used for genetic studies of several economically important crops [23,24,25,26,27,28]. In wheat, the construction of high-resolution genetic maps based on the microarray hybridisation-based technique called Diversity Arrays Technology (DArT) was developed. It enabled high-throughput genotyping without relying on sequence information [29]. Advancements in next-generation sequencing (NGS) technology have made it possible to screen for SNPs which have been used to develop high-throughput SNP-typing platforms such as BeadExpress [30], KASPar [31], Infinium [32], and designer SNP arrays [33]. A 9K SNP wheat chip detected genomic regions targeted by breeding and improvement selection [32,34]. Wang et al. [35] reported a 90K SNP iSelect array that comprises approximately 90,000 gene-associated SNPs with the potential to provide dense coverage of the wheat genome. This technology offers a resource for diversity studies and high-resolution dissection of complex traits in wheat.

The present study used a 90K iSelect array to survey SNPs across the mutation lines and the parent cultivar Chara. Data from the 90K analysis were compared with the whole wheat genome assembly to investigate the *1-FEH* gene mutations, map the mutation region in the chromosome, and annotate the mutational region. Accordingly, the response of each mutation line to WSC remobilisation and how that is influenced by terminal water stress was characterised. The knowledge generated from this research regarding the specific roles of the *1-FEH* gene isoforms provides the gene target for wheat breeding, particularly post-anthesis water-deficit conditions.

## 2. Materials and Methods

### 2.1. Plant Material

A set of mutation lines of the three isoforms of *1-FEH* (*1-FEH w1*, *w2*, and *w3*), previously developed in the Australian wheat cultivar Chara, was bulked up and used in this experiment (Table 1). Four germplasms were included in the study: one line each of *w1* mutant, *w2* mutant, and *w3* mutant was selected together with the parent Chara.

### 2.2. Backcrossing for 90K SNP Analysis

The mutated lines were backcrossed with the parental cultivar Chara in a Murdoch University glasshouse to check the mutation’s stability and reduce background genetic noise due to mutations elsewhere in the genome. The parent Chara was used as the female parent. The F1 seeds were collected on maturation at 65 days after pollination and were used to develop the iSelect 90K SNP array.

### 2.3. Genotypic DNA Extraction

DNA was extracted using an Illustra DNA Extraction Kit Phytopure (illustra Nucleon Phytopure Genomic DNA Extraction Kits, GE Healthcare, Amersham Place, Buckinghamshire, UK) following the standard protocol supplied by the manufacturer (Illustra Nucleon Phytoprue for small sample, 0.1 g RPN8510). One microlitre of extracted DNA was run on 1% agarose gel and standard lambda DNA (Axyzen Life Sciences, Arizona, AZ, USA) to check the quality. The DNA concentration was quantified using NanoDrop (ND 1000 Spectrophotometer, Thermo Fisher Scientific, Waltham, MA, USA). The DNA samples were then diluted to 20 ng µL^−1^ in 50 µL EB buffer (10 mM Tris-HCl pH 8.5) that stabilised the DNA.

### 2.4. The 90K SNP Chip Analysis

The Infinium 90K SNP iSelect platform was used for genotyping using BeadStation and iScan according to the manufacturer’s protocol from Illumina. Deletions in the HIB (Heavy Ion Bombardment) mutants were identified from output files using Illumina’s GenomeStudio v2011.1 (Illumina Inc., San Diego, CA, USA) and custom MS Excel macros and/or perl scrip, where shifts away from the euploid SNP cluster position indicate a deleted locus. Standard cluster files provided with Infinium products identify the expected intensity levels of genotype classes for each SNP. Raw intensity data for the three *FEH* HIBs and three *FEH* HIB × Chara backcrosses (as well as pre-existing data for Chara and other unrelated CSIRO HIBs) were loaded into Genomestudio, and the NormTheta (*x*-axis coordinate) and NormR (*y*-axis coordinate) values from the SNP cluster plots were exported. Shifts in cluster position were calculated between the euploid Chara and each mutant sample. The differences in Norm Theta and in NormR were calculated following the formula: Theta [Chara] − Theta [samples].

Shifts in cluster position were also calculated between the average HIB position (96 unrelated HIB mutants genotyped of Chara previously used in a different project and each of the *FEH* mutant samples). The 96 HIBs were used as an extra tool for helping to identify putative deletions due to the presence of Chara biotypes (i.e., different versions of ‘Chara’). Differences in Norma Theta and in Norma R were calculated using the formula: Theta [Av. HIB] − Theta [sample]. The 96 HIBs were used as an additional tool for helping identify deletions due to the presence of Chara biotypes (i.e., different versions of “Chara”). SNP loci putatively tracking deleted segments were identified in each FEH HIB mutant. SNPs with a minimum cluster shift between Chara and the HIB mutant of 0.1 along the Norm Theta axis were considered to be markers for deletion.

In order to help clarify the position of the *FEH* genes in chromosomes 6A, 6B, and 6D, sequences similar to the *FEH* gene sequence (FJ184991) were aligned in Gydle against the Chinese Spring genome assembly IWGSC. Regions containing 1-*FEH*-like sequences were identified from the alignments. SNPs were retained if they showed evidence for deletion and/or if they were mapped to 6A, 6B, or 6D. Markers considered as flanking deleted segments were identified for each HIB line by looking at the shift in cluster position. All SNPs were considered, even those with cluster shifts <0.2 Norm Theta and/or <0.5 Norm R. These markers were visually assessed in GenomeStudio to assess polymorphisms relative to Chara. To validate that the deletion detected was in fact located on the subgenome of interest (e.g., 6A) rather than a homoeologoue (e.g., 6B or 6D), those single-site SNPs located near either end of the deletion were visually assessed in GenomeStudio. A ‘null’ allele, where there is no fluorescence due to deletion of the hybridisation site, was considered to be indicative of the deletion occurring on the subgenome.

### 2.5. The 90K SNP Data Analysis

For mapping and analysing the SNP data, UGENE—Integrated Bioinformatics Tools (http://ugene.net/06/04/2018, accessed on 15 July 2023), UniprotKB (http://www.uniprot.org/, accessed on 15 July 2023), and CLC Main Workbench—QIAGEN Bioinformatics were used. CDS sequences and exons of *1-FEHw1*, *w3*, and *w2* were used for blast analysis against the IWGSC chromosomes 6A, 6B, and 6D. The public RNAseq database exVIP (http://www.wheat-expression.com/, accessed on 9 October 2022) was used to study the gene expression of these gene copies. Sequence annotations, including GO terms and Pfam domain information along with structural predictions, were performed using the BioMart in EnsmblPlants (http://plants.ensembl.org/biomart/martview/, accessed on 15 July 2023).

### 2.6. Glasshouse Experimental Setup

The experiment was set up as a Randomised Complete Block Design (RCBD) with 3 factors (Line × Treatment × Sampling stage) and 3 replications (Table 1), resulting in a total of 324 pots. Pots were initially randomised following the DiGGer design of R statistical software. Pots were rotated every alternate day to ensure equal solar radiation to all plants. The potting mix was the same as that used to grow wheat in glasshouses at Murdoch University [10]. The average temperature thresholds for night and day were 15 °C and 25 °C, respectively, and for humidity, they were 80% and 50%, respectively. The day length was not controlled. Hence, the photoperiod changed with the season. The day lengths ranged from 12 h in June to 14 h in November. Plants were watered using an irrigation mat for 5 min each, 3 times daily before anthesis.

### 2.7. Imposing Water Deficit

The main stem was identified at the mid-tillering stage, and the flowering date was recorded for every plant by tagging them on the first flowering date. When 50% of the plants were flowering, half were exposed to water deficit until grain maturity by turning off the water supplied via irrigation mats, and the other half were kept well-watered. In this stage, pots were arranged on four benches (two for water-deficit plants and two for well-watered plants). Weights of the unwatered pots were taken each second day to determine the extent of water loss, and the well-watered plants were weighed once a week to confirm the well-watered condition. Water was turned off to the well-watered pots when plants became yellow on maturity.

### 2.8. Sampling

The first sample was collected at the AR5 stage [36], which was followed by the second sample collected when the ear fully emerged, and the following six samplings for the preliminary and the main experiments were carried out at 4-day intervals after initiation of the water-deficit treatment (Table 1). The final sample was collected at maturity. Sampling for WSC analysis was undertaken between 11.00 and 17.00 h to study the shoot carbohydrate reserve accumulation and remobilisation pattern during grain filling.

At each sampling stage, three organs (the stem, leaf sheath, and blade) for each sample were collected from the main tiller of three plants of each pot as one sample and immediately put on dry ice. The remaining two plants were left for maturity and seed collection. Three biological replicates of each line were collected at each sampling stage. Samples were stored at −20 °C.

Harvest data were collected at maturity from the main stem and the tillers of 9 plants from 3 pots per line per treatment. The following parameters were recorded: kernel number (KN) per main spike, KN per plant, and spike number per plant. Grain was oven dried for 10 h at 60 °C, and grain weight per main spike, grain weight per plant, and thousand grain weight (TGW) of the main spike were recorded.

### 2.9. Carbohydrate Analysis

Samples for carbohydrate analysis were freeze dried (−50 °C) for 72 h and then oven-dried (70 °C) for 24 h. Frozen samples were chopped manually and ground using a tissue-lyser into less than 5 mm pieces in a cold room and manually divided into two parts. One part was stored at −80 °C for the RNA study, and the other was stored at −20 °C for WSC analysis. Carbohydrate analysis was performed on 170 mg of each ground sample using boiling deionised water for carbohydrate extraction followed by colorimetry using the anthrone reagent [10,37].

### 2.10. Calculation of WSC Remobilisation

The WSC concentration was highest at 12 DAA in all mutation lines and Chara. The WSC started declining from 12 DAA, and the rate of decline was elevated up to 22 DAA. Therefore, WSC remobilisation was calculated from 12 to 22 DAA.

WSC remobilisation rate (%dw × day^−1^) = (Stem WSC concentration (%dw) at 12 DAA − Stem WSC concentration (%dw) at 22 DAA)/10

### 2.11. Data Analysis

Analysis of variance was performed using IBM SPSS Statistics V21.0. Post hoc comparisons were conducted using Tukey’s Multiple Range Test at *p* < 0.05. Graphs were generated using Sigmaplot 13.0.

## 3. Results

### 3.1. Detecting the Mutation Region

From iSelect illumine 90K SNP array analysis, putative deletions were identified in the *FEH*-Heavy Ion Bombardment (HIB) mutations encompassing the *FEH* genomic regions (Table 2). The 6A mutants were larger deletions than the 6B and 6D ones. For each HIB line, markers at each end of the deletion were identified to estimate the size of the deletion.

SNPs with a minimum cluster shift between Chara and the HIB mutant of 0.1 along the Norm theta axis or 0.5 along the NormR axis were considered to be putative markers for a deletion. Deletions were confirmed by the Chara X mutant backcross, which were located at a ‘HET’ position, i.e., halfway between the mutant and the euploid Chara. The Infinium assay probes were bound to multiple sites in the genome due to the presence of homologues (e.g., 6A, 6B, and 6D) and paralogues, and polymorphism on any one of these sites was detected based on cluster position. The deletion of a segment of DNA in an HIB mutant affected the number of loci to which the probe could hybridise. This resulted in a shift in the NormTheta and/or NormR axes relative to the euploid sample, i.e., Chara (Supplementary Figure S1 as an example). When the probe had only one hybridisation site in the genome (e.g., hybridises to 6A but not 6B or 6D), the deletion of a hybridisation site resulted in a ‘null’ allele, i.e., a lack of fluorescent signal (Supplementary Figure S2 as an example). Those single-site SNPs, located near either end of the deletion, were visually assessed in Genome Studio. A ’null’ allele was considered to be indicative of the deletion occurring on the subgenome. Biotypes were observed between the Chara used to make HIBs and Chara used to backcross with the HIBs (Supplementary Figure S3 as an example).

### 3.2. Mapping of Mutation Regions in Chromosomes

High-density polymorphism clusters, considered to be mutated regions, were found on chromosomes 6A, 6B, and 6D, respectively (Table 2). By detecting the approximate genomic location of the mutation, the affected genes were subsequently identified and mapped. The deletion size was 28,246,961 bp (from 12,836,428 to 41,083,389 bp) on chromosome 6A (Supplementary Figure S4); 4,876,122 bp (from 54,419,374 to 59,295,496 bp) on chromosome 6B (Supplementary Figure S5) and 1,600,754 bp (from 28,682,554 to 30,283,308 bp) (Supplementary Figure S6) on chromosome 6D.

The entire list of genes in these intervals is provided in Supplementary Tables S1–S3.

### 3.3. Gene Annotation at the Mutated Region and Functional Analysis

The genes for both the targets and non-targets within the mutation regions of chromosomes 6A, 6B, and 6D were annotated with IWGSC Refseq v2.1 assembly using CLC Genomic Workbench. For the mutation region of *1-FEH w1*, 479 high-confidence (HC) genes were identified on chromosome 6A, including the *1-FEH w1* (Supplementary Table S1, Supplementary Figure S4), 90 HC genes were identified in the *1-FEH w3* mutation region on chromosome 6B including the *1-FEH w3* gene (Supplementary Table S2, Supplementary Figure S5) and 144 HC genes were identified in chromosome 6D’s mutated region along with *1-FEH w2* (Supplementary Table S3, Supplementary Figure S6). Functional annotation of the identified genes was carried out using the BioMart tool available on EnsemblPlants and by manually blasting against the IWGSC gene functional annotation V.1. A total of six, two, and five genes have been identified on the mutated regions of chromosome 6A, 6B and 6D, with similar function to the *1-FEH w1*, *1-FEH w3* and *1-FEH w2*, respectively. Further functional confirmation of uncharacterised non-target genes became essential to validate that the phenotypic change in the mutated plant is due to the deletion of the target genes, namely *1-FEHw1*, *w2*, *w3*. Thus, the function prediction of uncharacterised proteins was carried out following computational biology approaches. However, most of the genes were identified as encoding uncharacterised proteins based on sequence blasts in the available databases. To further predict the possible interference of the uncharacterised suite of genes with the function of *1-FEH*, gene expressional behaviours were compared from the available datasets at ExpVIP (http://www.wheat-expression.com, accessed on 9 October 2022). Results demonstrated that all of the genes had either very little or no expression compared to the corresponding *1- FEH* isoform at the reproductive stage (Supplementary Figures S7–S9).

Detailed interpretation of the functional properties of non-target genes indicated that none of them were directly related to *1-FEH w1*, *w2*, and *w3* gene functions (Table 3). So, it can be concluded that the phenotypic differences in the mutated lines in relation to WSC mobilisation can be considered to be due to the deletion of *1-FEH* isoforms.

### 3.4. Observation of the Water-Deficit Regime in Mutated Lines

**Figure 1:**
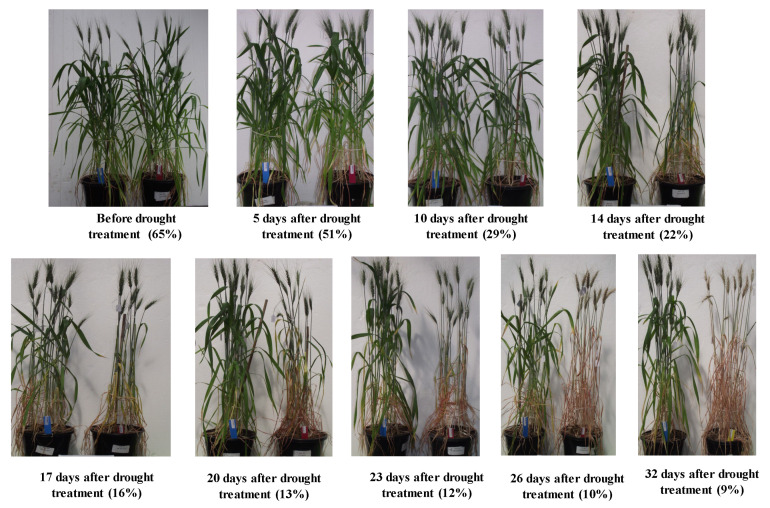
Effect of the water-deficit treatment on physical appearance of wheat plants over time. In each panel, the pot on the left-hand side is a well-watered reference, and the pot on right-hand side is the water-deficit treatment. The number as % under each panel is the soil moisture content of the water-deficit-treated pot. Appearance of wilted leaves and early leaf senescence were noted in the water-deficit-treated plants.

By 14 days after the water-deficit treatment was imposed, the soil moisture content had fallen to 22%, and plants showed signs of mild stress (first visible at 10 days) from leaf wilting. Plants reached the permanent wilting point (13% soil moisture content) after 20 days of water being withheld. Water deficit promoted early senescence compared to the well-watered plants (Figure 1).

### 3.5. Water Deficit Promoted Early Remobilisation of WSC

**Figure 2:**
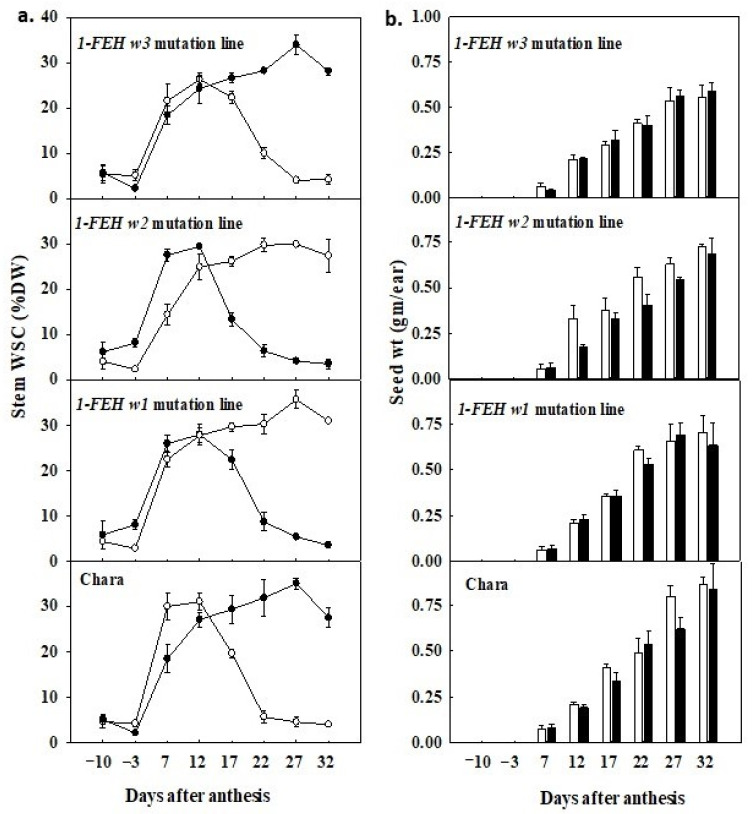
Change in stem water-soluble carbohydrate (WSC) concentration (**a**) and grain dry weight per ear with time after anthesis (**b**). Closed symbols and bars = well watered, and open symbols and bars = water deficit; from 10 days before anthesis to 32 days after anthesis, Bars with the same letter are not statistically different at *p* = 0.05 (n = 9), vertical bars indicate the ±SE of the mean of three replicates.

In well-watered plants, stem WSC levels peaked at 27 days after anthesis (DAA). There were no significant differences among parent and mutation lines (*w1*, *w2*, and *w3*) (Figure 2). In contrast, under water deficit, WSC peaked at 12 DAA, which is 15 days earlier than in the well-watered plants. Accordingly, the highest stem WSC level appeared significantly lower in water-deficit plants compared to the well-watered ones. In well-watered plants, the highest level of WSC was in Chara, with 35%, which was followed by *w3*, *w2*, and *w1* mutation lines with 33.9, 29.9, and 34.5%, respectively. On the other hand, in water-deficit plants, the highest level of stem WSC under water deficit was 31% in Chara followed by 29.9, 28.5, and 26.3% in w2, w1, and w3 mutation lines.

### 3.6. Timing of Decline in WSC Differed among Mutation Lines

**Figure 3:**
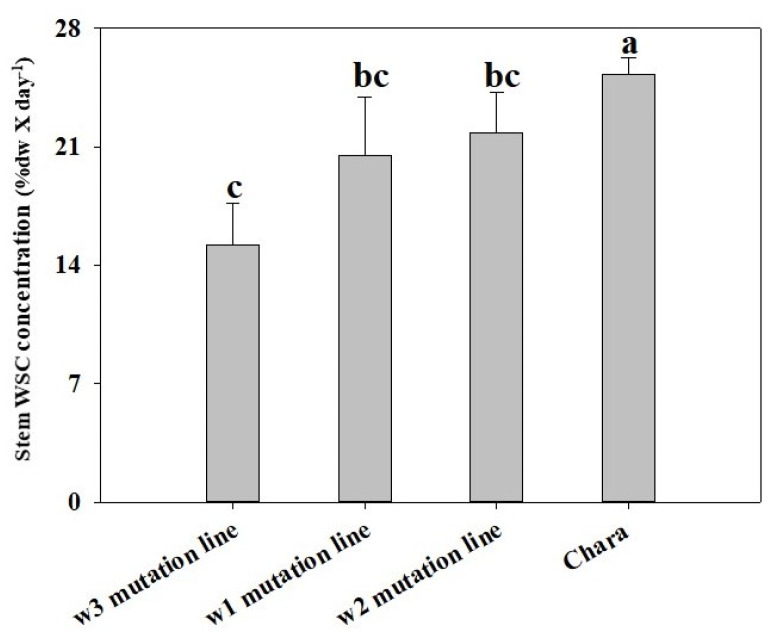
Decline in stem WSC (%DW) in three mutation lines and Chara under water deficit from 12 to 22 days after anthesis. Bars with the same letter are not statistically different at *p* = 0.05 (n = 9) vertical bars represent ±SE of the mean of three replicates.

There was a clear variation in the pattern of decline in WSC concentration in plants subjected to water deficit. The WSC remobilisation rate was slower in the *1-FEH w3* mutant line compared to Chara and the other two mutation lines. This was evident from the fact that the *1-FEH w3* mutation line maintained (at 22 DAA) a significantly higher (11.1%) level of stem WSC than Chara (5.7%, *p* < *0.07*), *1-FEH w2* (6.7%) and *1-FEH w1* (9.5%) mutant lines (Figure 2). However, there were no significant mean differences between Chara and the mutation lines in well-watered plants. Accordingly, under water-deficit conditions, between 12 DAA (peak value for WSC) and 22 DAA (permanent wilting point), the remobilisation rate of WSC was significantly (*p* < 0.05) lower in the *w3* mutation line (15.2%) compared to Chara (25.2%) and was slightly slower (not significantly) than mutation lines *w2* (21.8%) and *w1* (20.5%) (Figure 3).

### 3.7. WSC Concentration and Grain Development

There was a negative correlation between stem WSC concentration and developing grain weight under water deficit (between 12 and 32 DAA), but it was not the case in well-watered plants (Figure 2). In water-deficit plants, the WSC concentration began to decrease from 12 DAA, and the developing grain weight started to increase, indicating WSC was being remobilised to the grain. Even though at 12 DAA, there was no significant difference in the main spike seed weight, by 32 DAA, the seed weight was significantly (*p* < 0.05) lower in the *1-FEH w3* mutation line (0.56 g/ear) compared to Chara (0.87 g/ear) (Figure 2). However, there were no significant differences in *1-FEH w2* and *1-FEH w1* mutation lines compared with Chara for these developmental stages.

### 3.8. Grain Weight at Maturity Was Influenced by 1-FEH Isoforms under Water-Deficit Condition

**Figure 4:**
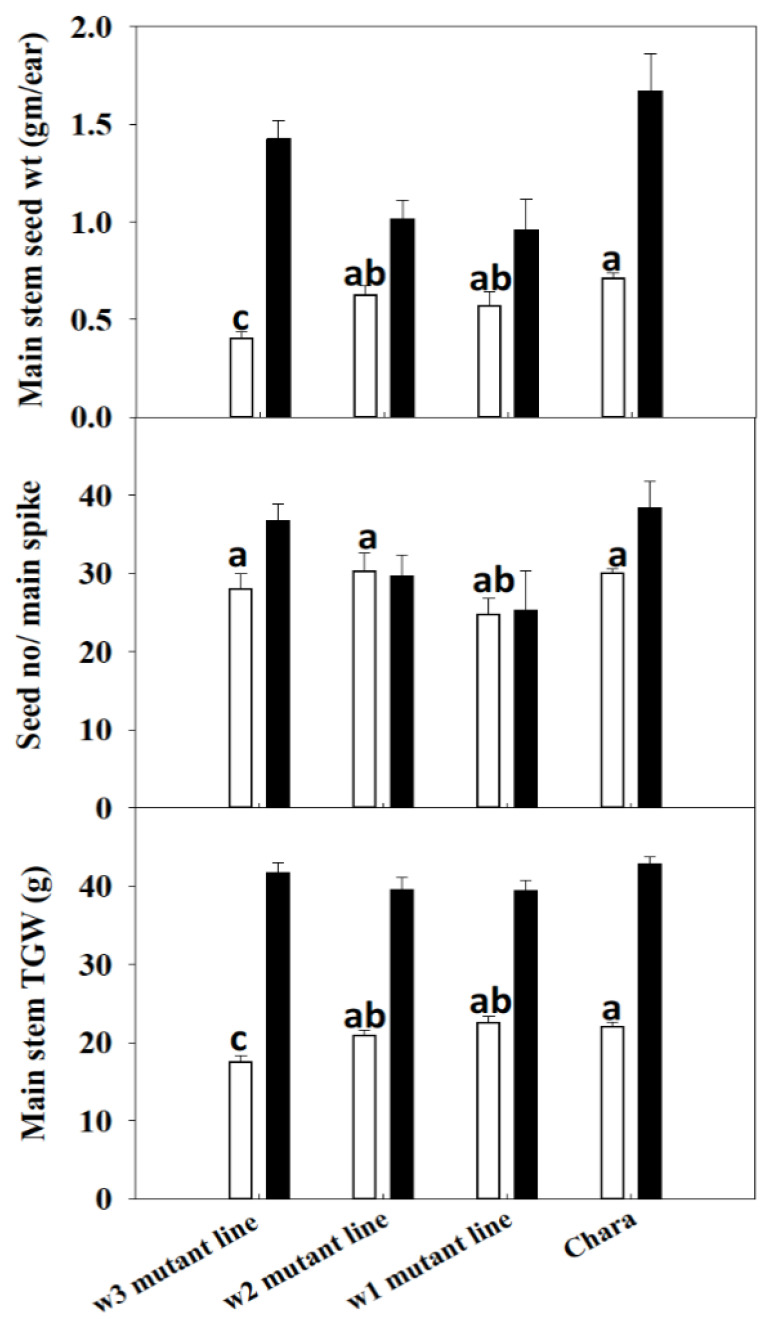
Main stem seed weight (g), seed no/spike and TGW at maturity in well-watered and water-deficit plants in all mutation lines and parent line Chara. Closed symbols = well-watered, and open symbols = water deficit; vertical bars represent ±SE of the mean of three replicates. Values with the same letter are not statistically different at *p* = 0.05 (n = 9) under water-deficit treatment.

Water deficit reduced (*p* < 0.05) the grain weight of the *1-FEH w3* mutant line compared to Chara and the other two mutation lines (Figure 4). Under water deficit, grain wt/main spike was the lowest in the *w3* mutation line (0.41 g ± 0.03) and the highest in Chara (0.71 g ± 0.03 SE). The other two mutation lines *1-FEH w2* and *1-FEH w1* produced 0.63 g ± 0.05 and 0.58 g ± 0.08 grain/main spike, respectively. In contrast, in the well-watered treatment, the *1-FEH w3* mutant line and Chara had similar seed wt/main spike (1.43 g ± 0.09 and 1.5 g ± 0.04, respectively) followed by *w2* (1.02 g ± 0.02) and *w1* (0.96 g ± 0.16). The TGW (thousand grain weight), a major determinant of the yield, was significantly lower (*p* < 0.05) in the *w3* mutation line (17.47 g) compared to Chara (22.53 g) under water deficit, but the *w2* and *w1* mutation lines did not show any significant difference with Chara. Under the well-watered condition, there were no significant differences among the mutation lines and Chara.

### 3.9. Correlation Analysis of Grain Weight with WSC Level and Remobilisation Rate

To understand potential factors influencing grain weight under water deficit, two correlation analyses were carried out: (1) main stem seed weight vs. the highest level of WSC; and (2) grain weight per main stem vs. WSC remobilisation rate. The correlation co-efficient of grain weight per main stem with sugar remobilisation rate was 0.78 (*r* = 0.78) (*p* < 0.01), while it was 0.66 (*r* = 0.66) (*p* < 0.05) (Supplementary Figure S10) in the case of the highest WSC level. Thus, grain weight per main stem was more strongly correlated with sugar remobilisation than the WSC level.

## 4. Discussion

### 4.1. Characterisation of 1-FEH Gene Mutations

Characterisation of the *1-FEH* mutation lines was carried out using high-density SNP genotyping array and bioinformatics approaches that provided detailed information regarding the physical modification of mutated segments on the chromosomes and the nature of the mutation. This information was crucial to explain the functional loss of the *1-FEH* genes in the mutation lines. It is important to note that the backcrossing step was necessary for the comparative analysis of mutants obtained from all mutagenesis screens, irrespective of the type of mutant identification strategy used, or type of mutagen used, or the organism used. Deletion mutations typically do not revert. To identify mutation regions, we focused on a high-density variation on a single chromosome for each mutant which corresponds to regions of high mutagen-induced damage that were not removed during backcrossing and therefore most likely genetically linked to the causal mutation [38]. We therefore focused our attention on these physical regions to identify candidate mutations. This approach offered the opportunity to compile and compare the parent line, mutation lines, and backcrossed lines and to identify the mutation region.

### 4.2. Chromosomal Mapping of Mutation Regions and Candidate Genes

To validate the phenotypic expression of gene function loss due to mutation, we characterised the structural changes that happened in the genes and adjacent regions of the chromosomes. The high-density SNPs clusters identified on chromosomes 6A, 6B, and 6D, respectively, clearly demonstrated the physical location of the mutated regions of the *1-FEH* mutation lines. The advancement of wheat whole genome sequencing [14] has furthermore enhanced the capacity for a detailed characterisation the chromosomal mutated regions by comparing the mutated line with the parent line. The sizes of the mutated/deleted regions were variable among the mutation lines. The largest deletion region was identified in the *1-FEH w1* mutation line, which was 28,246,961 bp on chromosome 6A. Deletion sizes of mutation lines *1-FEH w3* and *1-FEH w2* were 4,876,122 bp on chromosome 6B and 1,600,754 bp on chromosome 6D, respectively. Generally, mutation by heavy ion irradiation mutates a larger region compared to other ion radiation or chemical mutagenesis, and it can be highly variable. Knocking out *1-FEH* isoforms by using CRISPR-Cas9 genome editing technology or functional interference by using RNAi technology could provide the opportunity of precise and accurate investigation. However, a very high level of sequence similarity between *1-FEH* isoforms made it challenging to use these technologies. Moreover, several other members of the *FEH* gene family have high sequence similarities with *1-FEH* isoforms. These increase the possibility of off-target mutation/knockout using CRISPR-Cas9 or RNAi. 

### 4.3. Annotation of the Deleted Region

Mapping analysis demonstrated that the mutation affected significantly longer regions than the target gene regions of *1-FEH w1*, *w3*, and *w2* on chromosomes 6A, 6B, and 6D, respectively. Thus, annotating the whole mutated region was crucial to understanding the effect of deletion on the measured phenotypes. This analysis provided us with the functional information of target and non-target mutated genes. The investigation was also carried out to identify whether any non-target genes located within the mutated region influenced the target gene’s function.

A total of 379, 89, and 144 non-target high-confidence genes were identified in the mutated regions of chromosomes 6A, 6B, and 6D, respectively. These findings indicated that the mutation targeting the functional loss of *1-FEH w1*, *w2*, and *w3* affected a considerable number of genes at the adjacent chromosomal location.

### 4.4. Function Prediction for Non-Target Genes in Deleted Regions

Following the assembly of the mutation regions, further investigation on the functional annotation of non-target genes became essential to validate whether the phenotypic change in the mutated plant is due to the deletion of the target genes *1-FEH-w1*, *-w2*, *-w3*. This could be investigated by using either the bioinformatics approach or the molecular biology approach. With the release of wheat, whole genome sequences and robust gene annotation data, bioinformatics has become more reliable than ever. Moreover, the development of several powerful bioinformatic tools and databases has made this platform reliable to researchers as has been evident in the large number of published articles [14]. On the other hand, employing a molecular biology approach for the functional confirmation or expressional behaviour of large numbers of non-target genes is not feasible. Thus, this study used robust bioinformatic tools to confirm the functional behaviour of the non-target genes.

The IWGSC refseqv1.0 Functional Annotation v1 was the prime database used for the investigation of gene function. Furthermore, function prediction of non-targeted genes was carried out following computational biology approaches. The usual approach uses similarity-based searches within annotation databases. Several tools and databases were used to predict information about gene function using orthologue genes with high sequence similarity and proteins with significant structural homology.

The isoforms of *1-FEH* are hydrolase enzymes that catalyse the hydrolysis of chemical bonds. However, different hydrolase enzymes work on different kinds of bonds, meaning they break down different molecules. *1-FEHs* break down the *β*-(2-1) linkage of fructan, which is a storage carbohydrate in cereals. From the annotation, we identified *1-FEH w1*, *w2* and *w3* in the mutated regions of chromosome 6A, 6D and 6B, respectively, whose main functions are breaking down the sugar bond. Within the deletion regions of chromosomes 6A, 6B, and 6D, besides *1-FEH w1*, *w3*, and *w2*, another 5, 1, and 4 glycoside hydrolase genes were identified. However, their expressional analysis across publicly available databases clearly demonstrated that their expression level was negligible compared to the isoforms of *1-FEH* (Supplementary Figures S4–S6). Thus, we considered it unlikely that these genes had any influence on the sugar transport system.

### 4.5. Influence of 1-FEH Gene Mutations on WSC Remobilisation

This study investigated the performance of *1-FEH* isoforms mutation lines in sugar remobilisation under water-deficit and well-watered conditions. Mutation lines demonstrated functional variation of the isoforms in WSC remobilisation and grain filling. WSC content in the stem was measured at different stages during the grain filling, since it is the direct way to monitor differences in the accumulation and mobilisation of stem WSC.

### 4.6. The 1-FEH Gene Mutations Showed Functional Variation Only under Water-Deficit Condition Not under Well-Watered Condition

The results of this study clearly demonstrated that the functional role of different isoforms of the *1-FEH* gene was influenced by water availability. Under well-watered conditions, there was no difference in the WSC concentration, remobilisation rate, and grain weights between the mutation lines compared to the parent Chara under well-watered conditions. However, a significant variation was observed in the WSC remobilisation rate and grain weights under water deficit. Thus, it is evident that the 1-FEH enzyme plays a significant role in stem WSC regulation in water-deficit plants, leading to an effect on grain weight. In contrast, the equal performance of mutation lines and the parent in well-watered plants indicates that the 1-FEH enzyme did not play a significant functional role in stem WSC accumulation or remobilisation under well-watered conditions. A previous study [39] indicated differences in these isoforms of *1-FEH* at the transcription level in water-deficit wheat. This study used the *1-FEH* mutation lines for the first time, which confirmed this observation at the phenotype level.

### 4.7. Under Water-Deficit Conditions, WSC Remobilisation Rate and Grain Filling Were Slowed Down by the Deletion of 1-FEH w3

Under water deficit, WSC remobilisation was slower in the *1-FEH w3* mutation line compared to *1-FEH w1* and *1-FEH w2* mutation lines and the parent Chara. In water-deficit plants, the stem WSC peaked at 12 DAA, indicating the starting point of WSC remobilisation. Generally, the stage when the concentration of WSC reaches the highest level is considered as the start of carbohydrate remobilisation, since the process leads to a decline in the WSC concentration [40]. On the other hand, plants reached the permanent wilting point at 22 DAA, which would have reduced metabolic activity due to the scarcity of water. Thus, the period between 12 and 22 DAA in this trial was the most crucial for WSC remobilisation under water deficit. Usually, seed development occurs over 6 weeks after anthesis, and during the first two weeks, only cell division takes place in the endosperm and relatively little dry weight is gained. But from two weeks after anthesis, starch and protein begin to accumulate rapidly in the kernel and the dry weight increases almost in a linear manner. This is the crucial time for accumulating most of the final kernel weight [41]. So, the remobilisation of WSC during the mid-grain filling stage is crucial to maintain grain weight [42].

Noticeably, compared with the parent Chara and the other two mutant lines, at 22 DAA, the stem WSC concentration remained the highest in the *1-FEH w3* mutation line (10% DW) despite the fact that it had the lowest stem WSC content (26.25%) at the peak at 12 DAA. This clearly indicates that the average remobilisation rate of WSC was the slowest in the *1-FEH w3* mutation line. Indeed, WSC remobilisation was 39.8% slower in the *1-FEH w3* mutation line than in Chara. In contrast, mutation lines *1-FEH w1* (18.8% slower) and *1-FEH w2* (13.6% slower) maintained the statistically non-significant differences in remobilisation as the parent Chara. So, it is clearly evident that *1-FEH w3* is playing an important role in the remobilisation of WSC under terminal water-deficit conditions. 

### 4.8. Components of Grain Yield Were Reduced in the 1-FEH w3 Mutation Line but Not in 1-FEH w2 or 1-FEH w1 Mutation Lines

The results indicated that the slow remobilisation rate of WSC in the *1-FEH w3* mutation line directly affected seed development and ultimately resulted in a significant decrease in the final yield. Accordingly, the higher remobilisation in the other two mutation lines (*1-FEH w1* and *1-FEH w2*) and the parent Chara, which carries the *1-FEH w3* wild type, helped to reduce the grain weight loss from water-deficit stress. This study clearly shows that wheat without *1-FEH w3* would be disadvantaged when grown under post-anthesis water deficit.

It is evident that the three isoforms of *1-FEH* do not play equal roles in stem WSC remobilisation under water deficit. The absence of *w1* and *w2* did not have any significant effect on WSC remobilisation and seed weight development compared to the parent Chara. However, the absence of *1-FEH w3* disrupts sugar transportation to the developing grain in water-deficit plants, resulting in lower seed weight. Chara and the mutation lines *1-FEH w1* and *1-FEH w2* had the *1-FEH w3* isoform, which helped them to minimise the yield loss due to water deficit, which agreed with the previous studies [43,44]. It is worth mentioning that the three isoforms of *1-FEH* could have an additive effect in the resulting phenotype. A further investigation by utilising single, double and triple mutant/s of *1-FEH* isoforms would be able to provide insight on the additive effect of *1-FEH* isoforms.

This study reported the individual functions of the three isoforms of the *1-FEH* gene by using gene mutation through heavy ion irradiations. An extensive amount of information from 90K SNP-based genotyping, a wide range of genomic and bioinformatic analyses, and a glasshouse-based drought experiment supported the functional difference of *1-FEH* isoforms. Future studies by overexpressing the individual isoforms in wheat or in the model plant species and employing extensive molecular biology approaches will provide a further level of functional confirmation of the isoforms in regard to the WSC remobilisation under terminal drought.

## 5. Conclusions

We used a gene mutation approach to investigate the individual function of the three isoforms of *1-FEH* in wheat. The 90K SNP-based characterisation of gene mutation demonstrated that the deleted regions on all three chromosomes were larger than the targeted genomic regions. However, gene functional prediction analysis confirmed that none of the non-target mutated genes had a significant influence on sugar transportation or remobilisation. An investigation of WSC remobilisation rates, grain-filling rate, and grain weight demonstrated that *1-FEH w3* plays the most crucial role in stem WSC remobilisation under post-anthesis water deficit compared to the other two isoforms.

## Figures and Tables

**Table 1:** Experimental details.

Lines	*w1* mutant line -BW22-M3-22-w1
	*w3* mutant line -BW18b-M3-426-w3
	*w2* mutant line -BW21-M3-433-w2
	Parent: Chara
Treatments	Terminal water deficit (water deficit imposed from anthesis (50% plants at the flowering stage) until maturity)
	Wellwatered
Sample collection growth stages	AR5 (Auricle distance 5 cm)
	Fully emerged spike
	7 days after anthesis
	12 days after anthesis
	17 days after anthesis
	22 days after anthesis
	27 days after anthesis
	32 days after anthesis
	At grain maturity

**Table 2:** Summary of deletions observed in each *1-FEH* mutant.

			6A	6A x C	6B	6B x C	6D	6D x C					
Chr	Size of the Deletion (bp)	Region Containing *FEH*-like Sequence	22	C X22	426	Cx 426	433	Cx 433	SNP Index	SNP Name	Position	Description of Observed Poly	Comment
6AS	28,246,961		D	H					76,092	wsnp_CAP8_c6680_3136899	1,28,36,428---1,28,36,621	Poly	SNP near top of del in 22
			D	H					3722	BobWhite_c5092_422	1,30,71,495-----1,30,71,594	Null	Null SNP near top of del in 22
		FEH_6A	D	H									
			D	H					71,378	Tdurum_contig42906_732	3,26,58,889----3,26,58,989	Null	Example of SNP located in FEH
			D	H	d	h	d	h	55,811	RAC875_c25556_1250	3,26,60,703---3,26,60,803	Poly	Example of SNP located in FEH
			D	H					1675	BobWhite_c23193_170	4,02,21,884---4,02,21,984	Poly	
			D	H					41,259	Kukri_c14765_1655	4,10,83,842---4,10,83,933	Poly	
			D	H					3378	BobWhite_c44549_83	4,10,83,289---4,10,83,389	Poly	
6BS	4,876,122				D	H			10,716	BS00074183_51	5,44,19,374---5,44,19,474	Null	Null SNP near top of del in 426
		FEH_6B			D	H							
			d	h	D	H	d	h	55,811	RAC875_c25556_1250	5,72,96,208---5,72,86,308	Poly	Example of SNP located in FEH
					D	H	.	.	75,083	tplb0055h14_483	5,92,95,396---5,92,95,496	Poly	SNP near bottom of del in 426
6DS	1,600,754		d	h			D	H	18,485	D_GBQ4KXB01B5NHZ_336	2,86,82,554---2,86,82,785	Poly	SNP near top of del in 433
		FEH_6D											
			d	h	d	h	D	H	55,811	RAC875_c25556_1250	3,02,82,758---3,02,82,858	Poly	Example of SNP located in FEH
		s	d	h	d	h	D	H	46,499	Kukri_c5531_358	3,02,83,208---3,02,83,308	Poly	SNP near end of del in 433. Located in FEH

Note: D (Red): deletion detected on target chromosome; d: deletion detected on homoeologous chromosome. H (Green): deletion detected on target chromosome in heterozygous condition, h: deletion detected on homoeologous chromosome in heterozygous condition. Highlighted (yellow) area showing position for targeted gene. Polymorphisms (poly) assign genomic changes resulting from HIB to being either nulls or a change in sequence.

**Table 3:** Functional classification of the proteins positioned within the mutated segment.

Major Classification	Chromosome 6A	Chromosome 6B	Chromosome 6D
Hydrolase	TraesCS6A02G048200 TraesCS6A02G042300 TraesCS6A02G042400 TraesCS6A02G060700 (**Fructan 1-exohydrolase w1** (Source: UniProtKB/Swiss Prot; Acc: Q84PN8)) TraesCS6A02G060800 TraesCS6A02G069000	TraesCS6B02G080800 TraesCS6B02G080700 **Fructan 1-exohydrolase w3** (Source: UniProtKB/Swiss-Prot; Acc: B6DZC8)	TraesCS6D02G066800 TraesCS6D02G064100 TraesCS6D02G064200 TraesCS6D02G064300 TraesCS6D02G064400 **Fructan 1-exohydrolase w2** (Source: UniProtKB/Swiss-Prot; Acc: Q84LA1)
Ligase	TraesCS6A02G067500 TraesCS6A02G067600 TraesCS6A02G067700 TraesCS6A02G067800		TraesCS6D02G065100 TraesCS6D02G065200
Oxidoreductase	TraesCS6A02G034100 TraesCS6A02G041900 TraesCS6A02G055700		TraesCS6D02G072600
Transferase	TraesCS6A02G059300 TraesCS6A02G059700 TraesCS6A02G066000 TraesCS6A02G026800 TraesCS6A02G037400 TraesCS6A02G039500 TraesCS6A02G051200 TraesCS6A02G051300 TraesCS6A02G059700 TraesCS6A02G066000	TraesCS6B02G079600 TraesCS6B02G080100	

## Data Availability

Data will be available on request.

## Supplementary Materials

The following supporting information can be downloaded at: https://www.mdpi.com/article/10.3390/cimb45080419/s1, Supplementary Tables S1–S3: List of HC genes in deleted region in 6A, 6B, and 6D. Supplementary Figures S1–S3: Examples of SNP detecting a deletion in all homeologues (Screenshot from GenomeStudio). Supplementary Figures S4–S6: Deletion regions on chromosomes 6A, 6B, and 6D. Supplementary Figures S7–S9: Expression comparison of 1-FEH w1, 1-FEH w3 and 1-FEH w2 with the functionally close genes in the mutated region on 6A, 6B and 6D. Supplementary Figure S10: Correlation between seed weight/main stem and WSC remobilisation and WSC concentration.
