# Supplementary material for: Wheat Water-Soluble Carbohydrate Remobilisation under Water Deficit by 1-FEH w3
Source: Curr Issues Mol Biol. 2023 Aug 11;45(8):6634–50. doi: 10.3390/cimb45080419 (PMC10453044; doi:10.3390/cimb45080419)
Supplement: Supplementary file 1 [file cimb-45-00419-s001.zip › Figures_Supplimentary-Nusrat.pdf]

Supplementary Figures

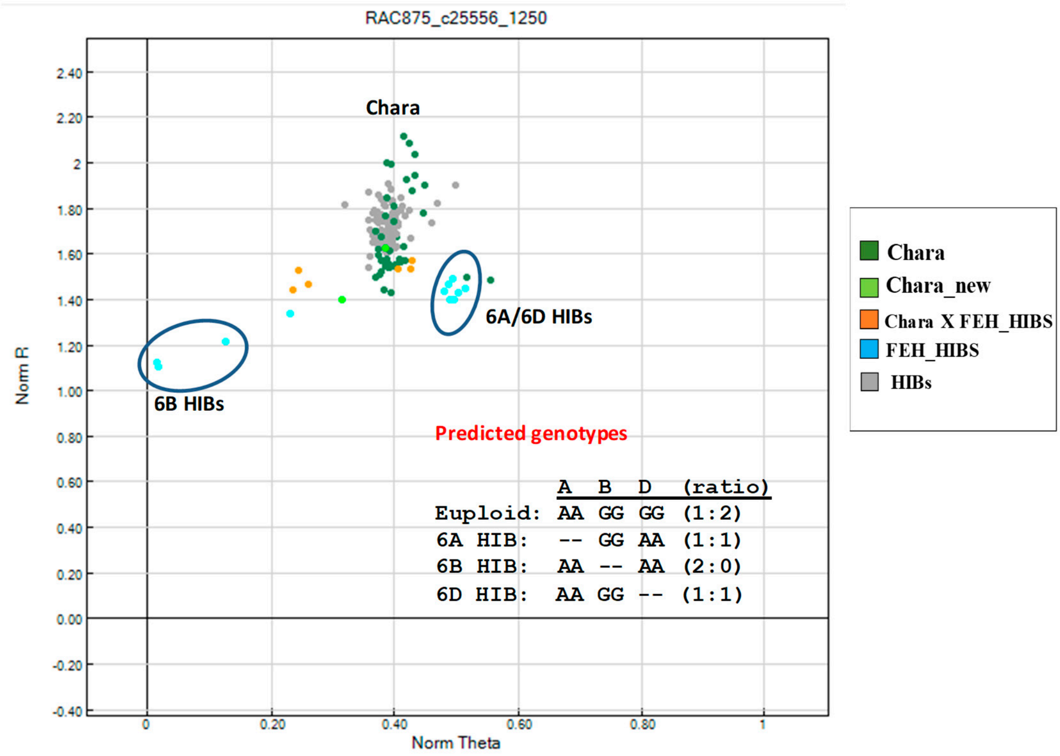

Supplementary Figure S1: Example of SNP detecting a deletion in all homeologues (SNP#55811). This SNP probe hybridised to a region within the *1-FEH* gene in all three homoeologues and detects a deletion in each HIB line. The genotypes are predicted based on shifts in NormaTheta (x-axis), which reflect the ratio of A/T to C/G alleles. [Screenshot from GenomeStudio]. Chara (dark green), provided Chara (light green), provided HIB mutants (light blue) and other HIBs genotype previously (light grey).

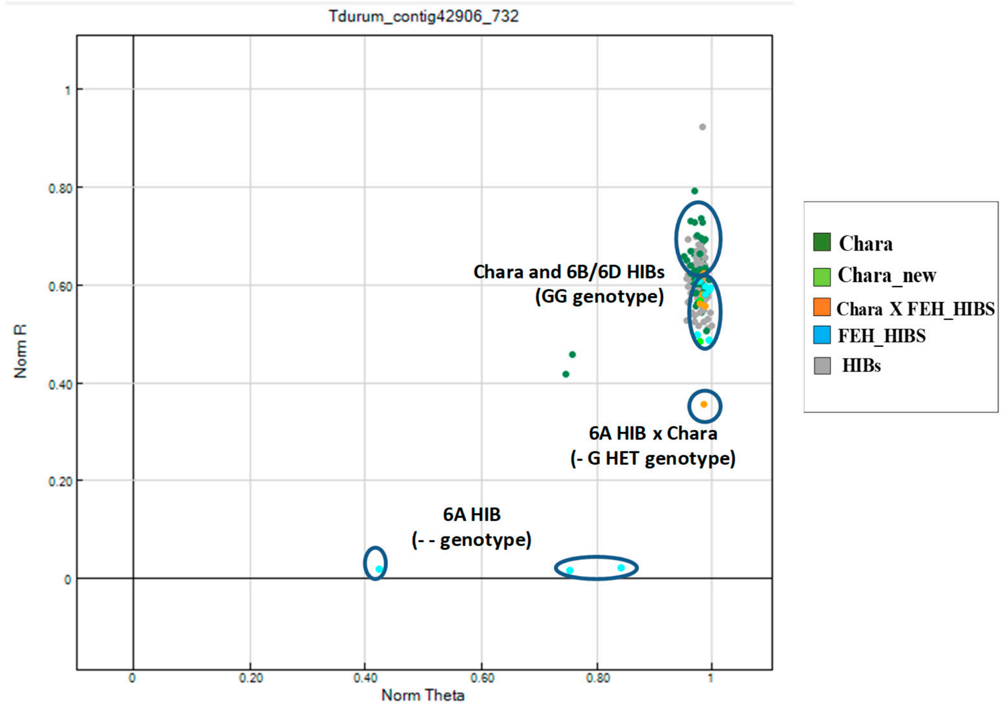

Supplementary Figure S2: Null allele for SNP#71378 in 6A HIB (Genome Studio Screenshot). Chara (dark green), provided Chara (light green), provided HIB mutants (light blue), and other HIBs genotyped previously (light grey).

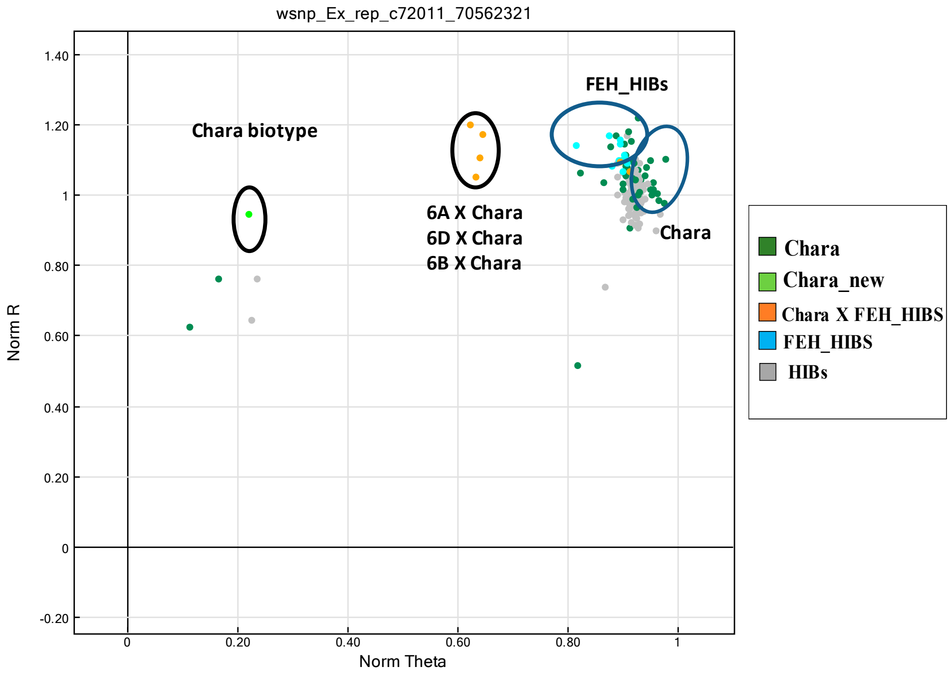

Supplementary Figure S3: Example of Chara biotype. The provided Chara (light green) is located in a different position compared to all provided HIB mutants (light blue) and other HIBs genotyped previously (light grey).

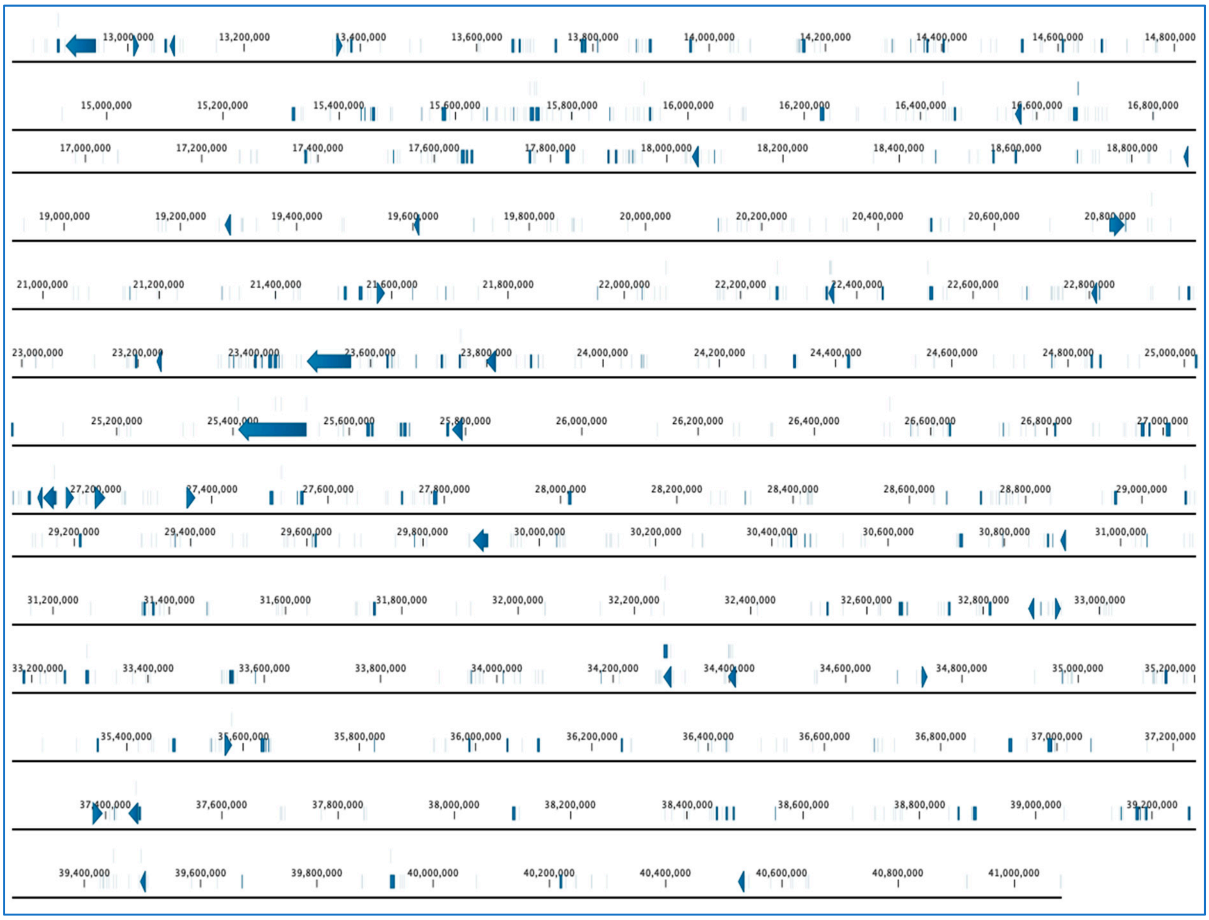

Supplementary Figure S4: Deletion region on Chromosome 6A.The dark blue bar/arrow indicates positions of high confidence genes and the light blue bar/arrow indicates positions of low confidence genes. The whole list of the genes is available in **supplementary Table S1**.

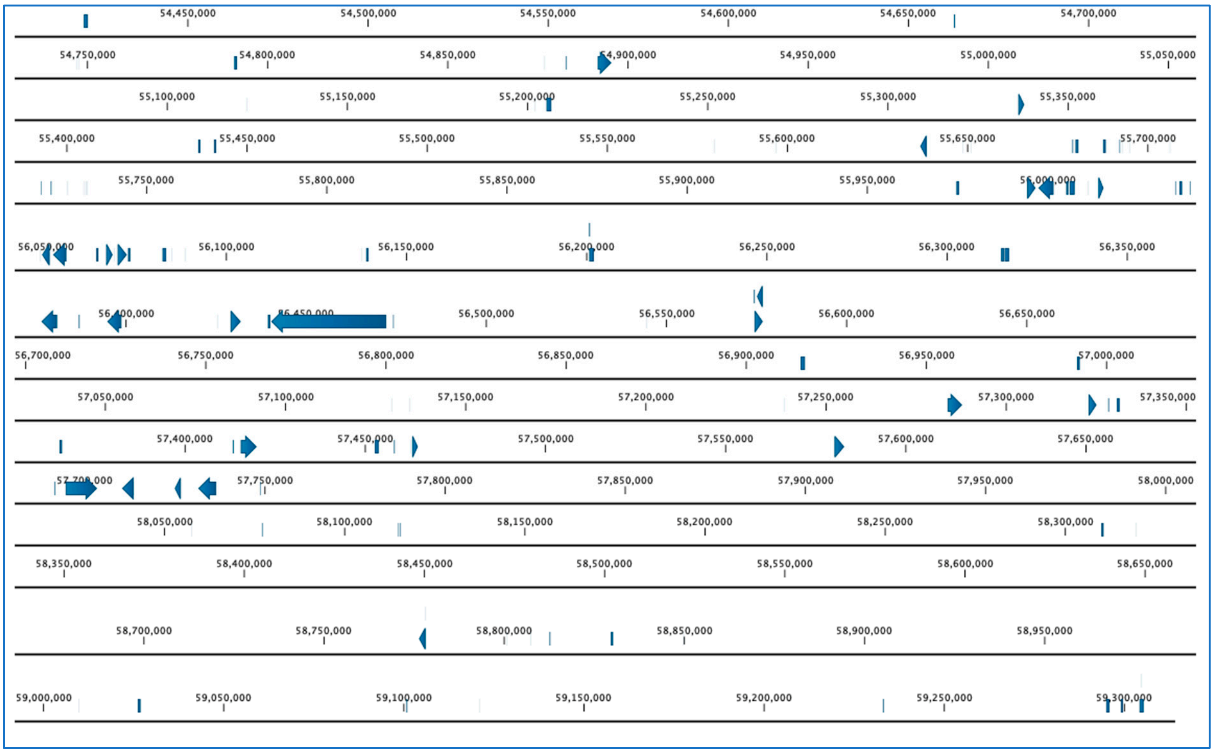

Supplementary Figure S5: Deletion region on Chromosome 6B. Dark blue bar/arrow indicate positions of high confidence genes and the light blue bar/arrow indicates positions of low confidence genes. The whole list of the genes is available in **supplementary Table S2**.

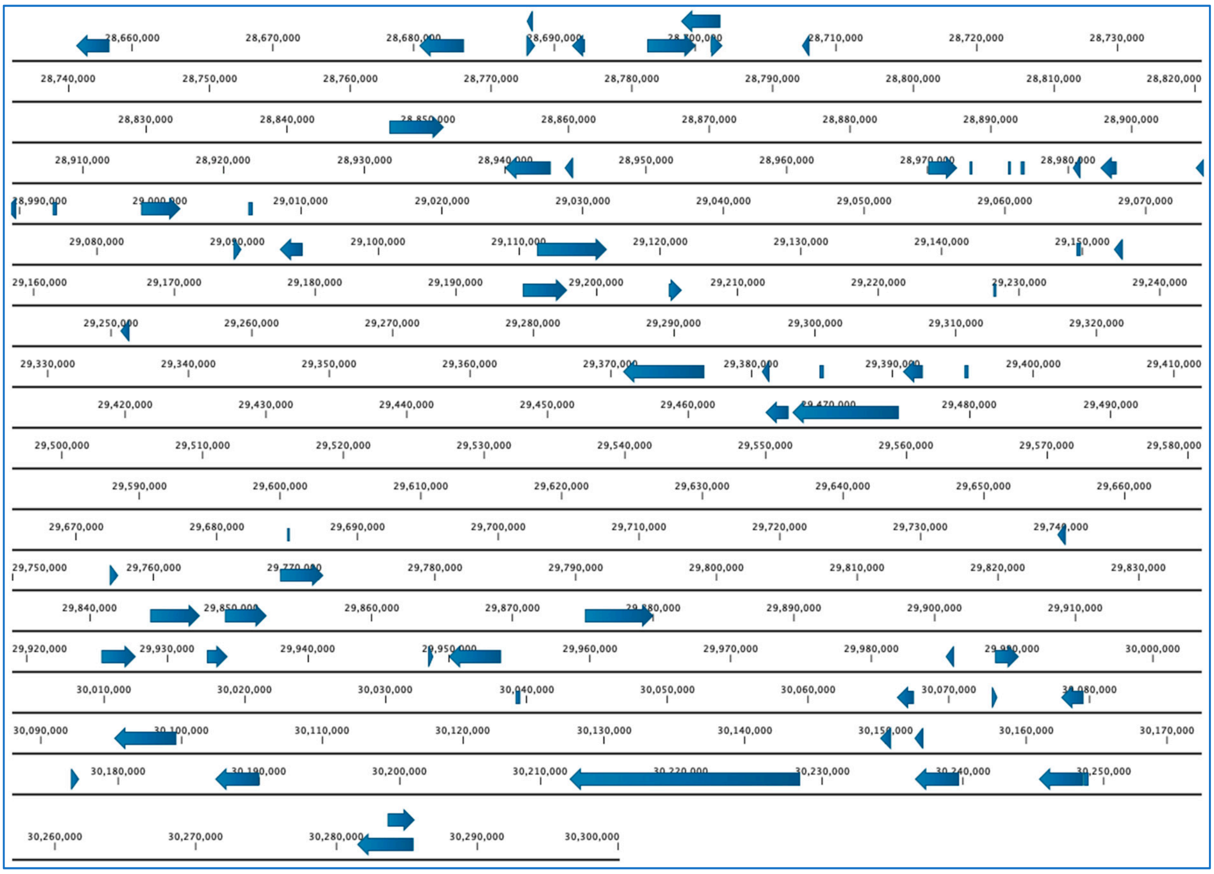

Supplementary Figure S6: Deletion region on Chromosome 6D. Dark blue bar/arrow indicate positions of high confidence genes and the light blue bar/arrow indicate positions of low confidence genes. The whole list of the genes is available in **supplementary Table S3**.

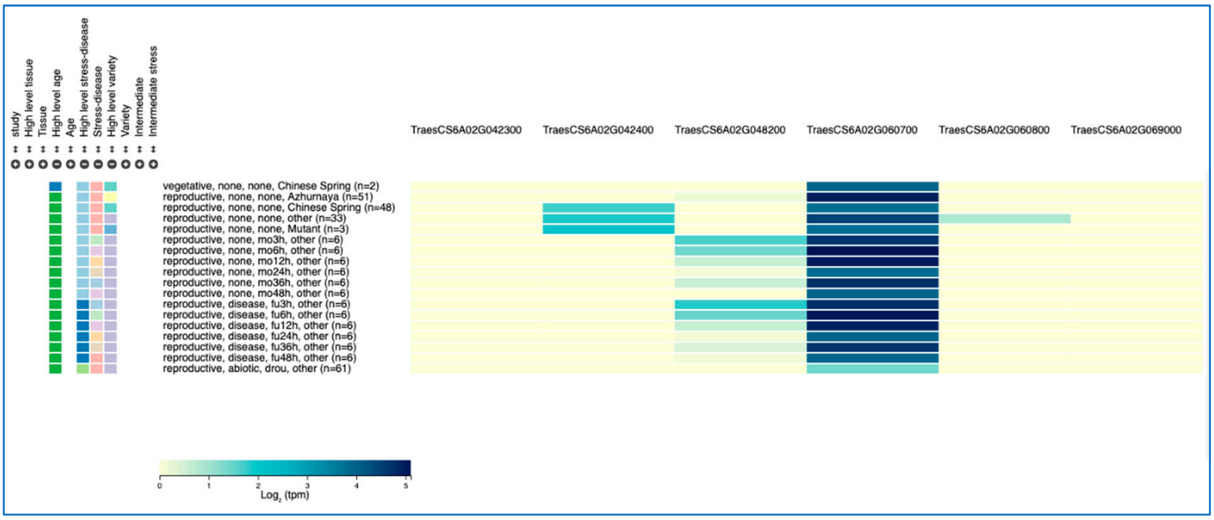

Supplementary Figure S7: Expressional comparison of 1-FEH w1 with the identified functionally close genes in the mutated region on 6A. Gene expressions were compared from all the available related datasets at ExpVIP (http://www.wheat-expression.com). No of samples used in expression analysis has been indicated as "n" in each of the individual experiments.

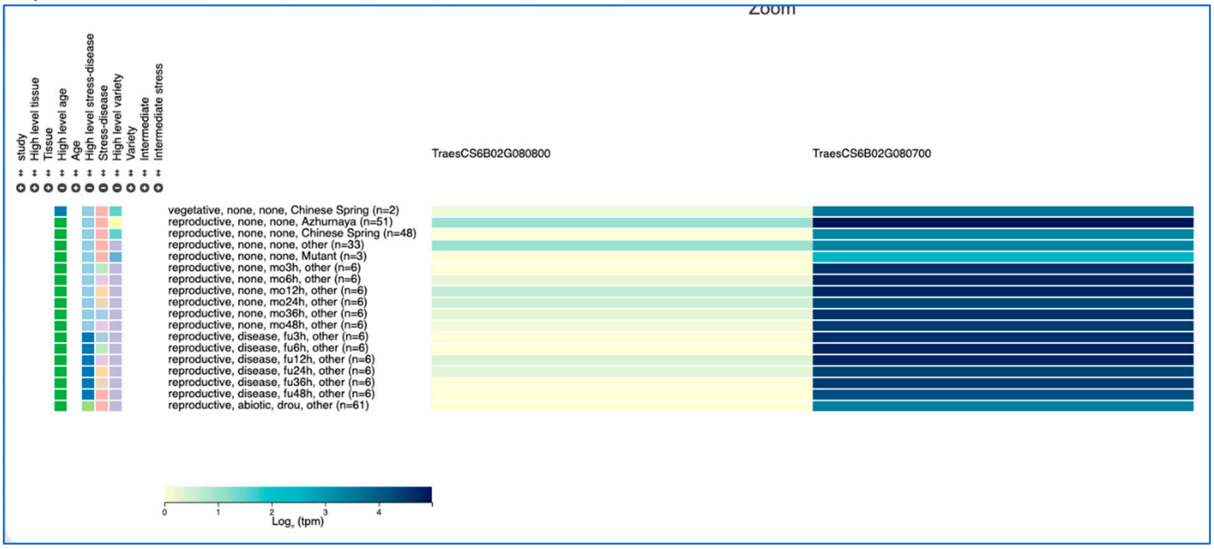

Supplementary Figure S8: Expressional comparison of 1-FEH w3 with the identified functionally close genes in the mutated region on 6B. Gene expressions were compared from all the available related datasets at ExpVIP (http://www.wheat-expression.com). No of samples used in expression analysis has been indicated as "n" in each of the individual experiments.

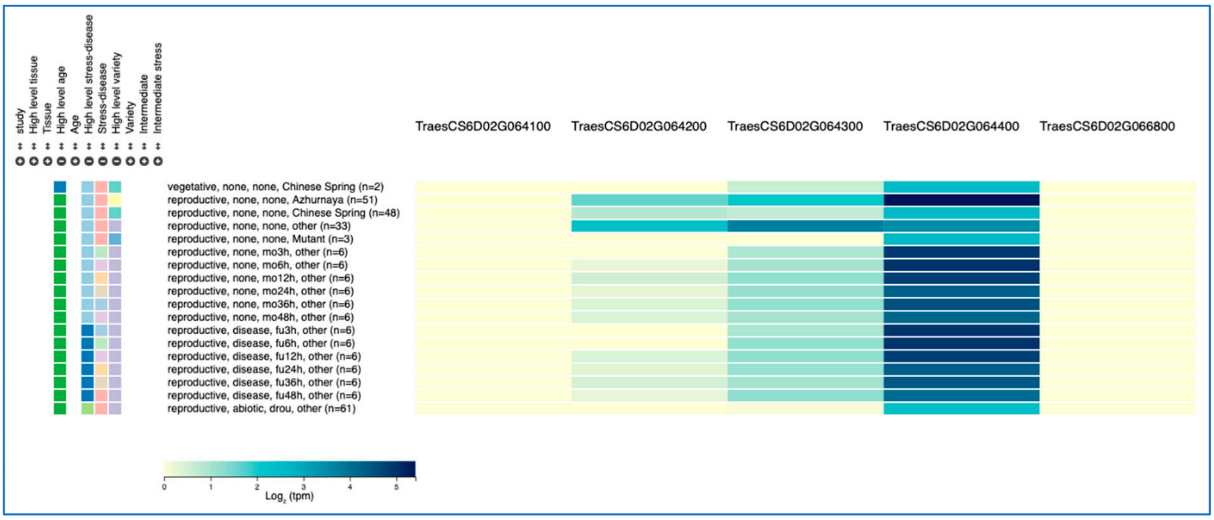

Supplementary Figure S9: Expressional comparison of 1-FEH w2 with the identified functionally close genes in the mutated region on 6D. Gene expressions were compared from all the available related datasets at ExpVIP (http://www.wheat-expression.com). No of samples used in expression analysis has been indicated as “n” in each of the individual experiments.

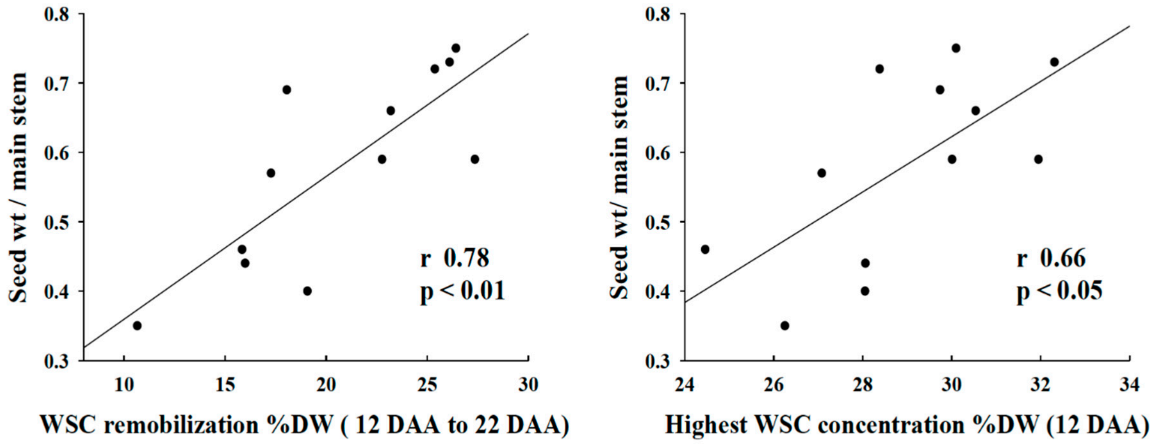

Supplementary Figure S10: Correlation with seed wt/main stem with WSC remobilization and WSC concentration.
